# Supplementary material for: Taxonomic Composition and Diversity of the Gut Microbiota in Relation to Habitual Dietary Intake in Korean Adults
Source: Nutrients. 2021 Jan 26;13(2):366. doi: 10.3390/nu13020366 (PMC7912254; doi:10.3390/nu13020366)

Supplementary material

Article title: Taxonomic Composition and Diversity of the Gut Microbiota in Relation to Habitual Dietary Intake in Korean Adults

Journal name: Nutrients

Hwayoung Noh [1,2,†], Hwan-Hee Jang [3,†], Gichang Kim [3], Semi Zouiouich [1], Su-Yeon Cho [3], Hyeon-Jeong Kim [3], Jeongseon Kim [4], Jeong-Sook Choe [3], Marc J. Gunter [1], Pietro Ferrari [1], Augustin Scalbert [1] and Heinz Freisling [1,*]

[1] Section of Nutrition and Metabolism, International Agency for Research on Cancer (IARC-WHO), 69008 Lyon, France; hwayoung.noh@inserm.fr (H.N.); ZouiouichS@students.iarc.fr (S.Z.); GunterM@iarc.fr (M.J.G.); FerrariP@iarc.fr (P.F.); ScalbertA@iarc.fr (A.S.)

[2] Department of Cancer Prevention and Environment, INSERM UA8, Léon Bérard Cancer Center, 69003 Lyon, France

[3] National Institute of Agricultural Sciences, Rural Development Administration (NAS-RDA), Wanju, Jeollabuk-do 55365, Korea; rapture19@korea.kr (H.-H.J.); recall@korea.kr (G.K.); 0727jsy@naver.com (S.-Y.C.); mamimumemo14@hanmail.net (H.-J.K.); swany@korea.kr (J.-S.C.)

[4] Department of Cancer Biomedical Science, Graduate School of Cancer Science and Policy, National Cancer Center, Goyang, Gyeonggi-do 10408, Korea; jskim@ncc.re.kr

* Correspondence: FreislingH@iarc.fr; Tel.: +33-472-738-664

† These authors equally contributed to this work.

This document contains the following items:

Table S1: Food group classification

Table S2: Difference of relative abundance (% OTU) of four major phyla and *Firmicutes-to-Bacteroidetes* (*F/B*) ratio by general characteristic and lifestyle factors of Korean adults of the NAS-IARC cross-sectional study (n=222 participants)

Table S3: Associations of within-subject (α-) and between-subject (β-) diversity of the gut microbiota with general characteristics and lifestyle factors of Korean adults of the NAS-IARC cross-sectional study (n=222 participants)

Table S4: Spearman correlations between the within-subject (α-) diversity of the gut microbiota and dietary intake of foods and nutrients in Korean adults of the NAS-IARC cross-sectional study (n=222 participants)

Table S5: Factor loadings of each food group for high α-diversity dietary patterns (HiαDPs) based on three α-diversity indices in Korean adults of the NAS-IARC cross-sectional study (n=222 participants)

Table S6: Spearman correlations between the high α-diversity dietary pattern (HiαDP) and the relative abundance of *Firmicutes* and its genera in Korean adults of the NAS-IARC cross-sectional study (n=222 participants)

Table S7: Spearman correlations between intakes of main contributing food groups of the high α-diversity dietary pattern (HiαDP) and the relative abundance of genera within the *Bacteroidetes* and *Firmicutes* in Korean adults of the NAS-IARC cross-sectional study (n=222 participants).

Table S8: General characteristics and lifestyle factors by enterotypes in Korean adults of the NAS-IARC cross-sectional study (n=222 participants)

Figure S1: Plots to select optimal numbers of clusters with Elbow, Silhouette, and Gap statistic methods on k-means clustering for identifying enterotypes based on unweighted and weighted UniFrac, and Bray-Curtis distance matrices.

Figure S2: Enterotypes of gut microbiota in Korean adults of the NAS-IARC cross-sectional study (n=222 participants) based on unweighted UniFrac.

Table S1. Food group classification

Food groups	Food items
Potatoes	Potato and sweet potato
Vegetables	Vegetable, vegetable juice, and mushrooms
Fermented vegetables	Kimchi and pickled vegetables
Seaweeds	laver, sea mustard, and kelp
Legumes	Legumes and their products (e.g. tofu, soy milk)
Fermented legumes	Fermented soybeans
Fruit/Fruit juice	Fresh or canned fruit and fruit juice
Nuts/Seeds	Almond, peanuts, sesame, etc.
Dairy products	Milk, cheese, ice creams, etc.
Refined grains	Refined rice
Multi/Whole grains	Whole-grain rice and other grains
Other cereal products	Noodle, rice cakes (non-sweet), breakfast cereals
Meats	Beef, pork, poultry, etc.
Processed meats	Ham and sausage
Fish/Seashells	Fish, coleoids, shellfish, etc
Eggs	Chicken/Quail eggs
Vegetable oils	Corn oil, sesame oil, etc.
Other fats	Butter, margarine, etc.
Sugar/Confectionary	Sugar, honey, jam
Cakes/Sweets	Cakes, rice cakes (sweet), pie, sweet biscuits
Coffee	Coffee
Tea	Green tea
Other non-alcoholic beverage	Carbonated or sweet beverages
Pizza/burgers	Pizza and burgers
Salty snacks	Chips and salty biscuits

Table S2. Difference of relative abundance (% OTU) of four major phyla and *Firmicutes-to-Bacteroidetes* (*F/B*) ratio by general characteristics and lifestyle factors of Korean adults of the NAS-IARC cross-sectional study (n=222 participants)

	Bacteroidetes		*Firmicutes*		*Proteobacteria*		*Actinobacteria*		*F/B ratio*	
	Median	IQR[a]	Median	IQR[a]	Median	IQR[a]	Median	IQR[a]	Median	IQR[a]
Total subjects (n=222)	54.2%	40.7-65.5%	37.6%	26.3-49.8%	3.8%	1.9-7.4%	0.39%	0.11-1.22%	0.68	0.41-1.22
Sex										
Men (n=108)	54.3%	44.1-67.1%	32.8%	23.8-46.8%	5.1%	2.8-10.7%	0.40%	0.14-1.20%	0.61	0.35-1.07
Women (n=114)	54.1%	39.4-63.2%	39.8%	30.5-51.4%	3.0%	1.5-5.5%	0.37%	0.12-1.24%	0.73	0.48-1.31
p-value[b]	0.133		0.009		<.001		0.947		0.024	
Age group										
<40 years (n=175)	53.7%	41.3-65.9%	36.3%	25.3-49.1%	4.1%	2.3-7.9%	0.42%	0.14-1.17%	0.63	0.39-1.21
≥ 40 years (n=47)	54.8%	39.4-63.7%	39.9%	28.5-51.7%	2.9%	1.4-5.7%	0.36%	0.08-1.38%	0.74	0.48-1.31
p-value[b]	0.446		0.128		0.025		0.921		0.203	
BMI group										
<23 kg/m^2 (n=128)	52.9%	40.8-62.9%	37.6%	26.8-50.5%	4.2%	1.9-7.5%	0.38%	0.09-1.27%	0.69	0.42-1.24
≥23 kg/m^2 (n=94)	56.2%	40.7-67.2%	37.5%	26.1-48.2-%	3.6%	1.9-6.6%	0.42%	0.19-0.97%	0.64	0.39-1.16
p-value[b]	0.179		0.507		0.371		0.938		0.343	
Dietary supplement intake										
Yes (n=76)	54.9%	42.1-63.7%	37.7%	27.4-50.2%	2.8%	1.3-5.9%	0.31%	0.08-0.96%	0.67	0.43-1.23
No (n=143)	53.3%	40.5-66.1%	37.7%	26.0-50.1%	4.3%	2.5-7.4%	0.51%	0.19-1.22%	0.68	0.39-1.25
p-value[b]	0.926		0.914		0.015		0.146		0.972	
Regular physical activity										
Yes (n=93)	52.3%	39.4-63.8%	37.8%	28.2-50.1%	3.7%	1.9-6.6%	0.39%	0.10-1.16%	0.70	0.44-1.31
No (n=129)	55.7%	41.3-65.5%	36.5%	25.3-49.1%	4.0%	2.2-7.4%	0.41%	0.15-1.22%	0.65	0.38-1.16
p-value[b]	0.368		0.415		0.844		0.411		0.318	
Smoking status										
Ever (n=54)	57.4%	47.8-69.9%	33.5%	23.8-45.9%	3.5%	2.4-7.4%	0.37%	0.12-1.17%	0.63	0.35-0.99
Never (n=168)	53.2%	39.1-63.5%	38.3%	27.7-51.1%	3.9%	1.7-7.4%	0.42%	0.13-1.25%	0.70	0.43-1.32
p-value[b]	0.017		0.075		0.873		0.391		0.040	
Education										
<University graduation (n=110)	55.9%	40.2-65.9%	34.1%	26.1-48.2%	4.7%	2.3-8.7%	0.50%	0.19-0.99%	0.62	0.39-1.22
≥University graduation (n=113)	53.0%	42.0-64.9%	40.0%	27.2-50.5%	3.5%	1.7-5.9%	0.33%	0.10-1.28%	0.73	0.42-1.23
p-value[b]	0.893		0.234		0.067		0.299		0.401	
Household Income										
<4,000 USD/month (n=79)	53.0%	43.8-65.5%	35.9%	24.5-50.1%	3.7%	1.5-5.9%	0.39%	0.19-0.92%	0.64	0.39-1.19
≥4,000 USD/month (n=93)	55.3%	40.2-66.1%	37.9%	27.0-48.6%	3.4%	1.8-6.6%	0.36%	0.08-1.16%	0.68	0.39-1.23
p-value[b]	0.905		0.649		0.761		0.690		0.660	

[a.] as interquartile range

[b.] The differences of % OTU of major four phyla and the *F/B* ratio by basic characteristic and lifestyle factors were examined by Wilcoxon-Mann-Whitney test

Table S3. Associations of within-subject (α-) and between-subject (β-) diversity of the gut microbiota with general characteristics and lifestyle factors of Korean adults of the NAS-IARC cross-sectional study (n=222 participants)

	α-diversity[a,b]						β-diversity[c]					
	Chao1		Shannon		Faith PD		Unweighted UniFrac		Weighted UniFrac		Bray-Curtis	
	F statistic	*p*-value	F statistic	*p*-value	F statistic	*p*-value	F statistic	*p*-value	F statistic	*p*-value	F statistic	*p*-value
Sex	1.08	0.300	6.99	0.009	3.33	0.069	2.25	0.002	2.06	0.100	2.29	0.032
Age group	4.92	0.028	10.38	0.002	10.47	0.001	2.70	0.001	1.11	0.310	2.29	0.026
BMI group	0.26	0.613	1.59	0.208	0.03	0.865	1.03	0.326	3.14	0.026	2.66	0.007
Dietary supplement intake	0.28	0.599	0.34	0.561	0.34	0.560	1.18	0.165	0.40	0.783	1.12	0.314
Physical activity	4.96	0.027	1.33	0.250	5.11	0.025	1.39	0.054	0.60	0.593	0.60	0.841
Smoking status	0.02	0.887	4.05	0.046	0.95	0.331	1.18	0.148	2.90	0.047	2.00	0.045

Age group (≥ 40 *vs*. <40 years); BMI group (<23 kg/m^2 *vs*. ≥23 kg/m^2); Dietary supplement intake within 3 month (yes *vs*. no); Regular physical activity (yes *vs*. no); Smoking status (ever *vs*. never)

[a.] The α-diversity indices were log-transformed

[b.] The statistical significance of associations of the α-diversity with basic characteristic and lifestyle factors were tested by general linear models (GLM)

[c.] The statistical significance of associations of the β-diversity with basic characteristic and lifestyle factors were tested by permutational multivariate analysis of variance (PERMANOVA)

Table S4. Spearman correlations[a] between the within-subject (α-) diversity of the gut microbiota and dietary intake of foods and nutrients in Korean adults of the NAS-IARC cross-sectional study (n=222 participants)

	α-diversity[b]					
	Chao1		Shannon		Faith PD	
	Coefficient	*p*-value	Coefficient	*p*-value	Coefficient	*p*-value
Food groups[c]						
Potatoes	0.04	0.564	0.20	0.004	0.04	0.554
Vegetables	0.01	0.897	0.20	0.004	0.01	0.879
Fermented vegetables	-0.03	0.713	0.07	0.298	-0.02	0.779
Seaweeds	-0.09	0.180	0.15	0.033	-0.10	0.145
Legumes	-0.05	0.453	0.09	0.185	-0.04	0.516
Fermented legumes	0.09	0.214	0.20	0.003	0.10	0.150
Fruit/Fruit juice	-0.06	0.398	0.13	0.064	-0.01	0.857
Nuts/Seeds	0.07	0.339	0.13	0.054	0.06	0.415
Dairy products	-0.04	0.580	0.08	0.244	-0.02	0.760
Refined grains	-0.06	0.382	-0.05	0.467	-0.08	0.236
Multi/Whole grains	0.02	0.781	-0.01	0.849	-0.01	0.895
Other cereal products	-0.08	0.260	-0.08	0.232	-0.03	0.668
Meats	0.06	0.385	0.04	0.546	0.03	0.655
Processed meats	0.02	0.789	-0.02	0.726	0.04	0.597
Fish/Seashells	0.05	0.499	0.10	0.143	0.04	0.575
Eggs	-0.04	0.599	0.03	0.693	-0.02	0.776
Vegetable oils	0.01	0.851	0.08	0.268	0.05	0.429
Other fats	0.04	0.566	0.04	0.597	0.00	0.951
Sugar/Confectionary	0.04	0.535	0.07	0.287	0.05	0.477
Cakes/Sweets	-0.07	0.341	0.06	0.412	0.00	0.981
Coffee	0.07	0.313	0.07	0.283	0.03	0.638
Tea	0.14	0.048	0.10	0.163	0.11	0.094
Other non-alcoholic Bev.	-0.02	0.750	-0.11	0.111	-0.04	0.523
Pizza/burgers	0.01	0.880	0.07	0.334	0.05	0.506
Salty snacks	-0.04	0.581	0.05	0.467	-0.03	0.681
Macronutrients[c]						
Plant protein	0.00	0.950	0.13	0.065	0.02	0.736
Animal protein	0.04	0.571	0.10	0.134	0.02	0.717
Carbohydrate	-0.09	0.171	-0.02	0.784	-0.10	0.144
Dietary fiber	-0.03	0.653	0.19	0.004	0.00	0.994
Plant fat	0.00	0.969	0.06	0.401	0.03	0.618
Animal fat	0.03	0.628	0.06	0.366	0.02	0.788
SFA	0.00	0.991	0.04	0.522	0.00	0.982
MUFA	0.02	0.736	0.05	0.497	0.01	0.842
PUFA	0.02	0.799	0.10	0.147	0.04	0.514
Alcohol	0.02	0.723	0.03	0.645	0.00	0.990

Faith PD, Faith's phylogenetic diversity

[a.] Partial Spearman correlation analysis with adjustment for sex, age, BMI, dietary supplement intake, physical activity, smoking status, and batch effect

[b.] The α-diversity indices were log-transformed

[c.] The intakes of food groups and macronutrients were log-transformed and adjusted for total energy intake using the residual method

Table S5. Factor loadings of each food group for high α-diversity dietary patterns (HiαDPs)[a] based on three α-diversity indices in Korean adults of the NAS-IARC cross-sectional study (n=222 participants)

	HiαDPs		
	Chao1[b]	Shannon[b]	Faith PD[b]
Food groups[c]			
Fermented legumes	0.42	0.41	0.38
Potatoes	0.23	0.33	0.25
Vegetables	0.27	0.33	0.24
Seaweeds	-0.03	0.32	0.00
Nuts/Seeds	0.36	0.26	0.30
Tea	0.32	0.25	0.29
Fruit/Fruit juice	0.03	0.24	0.18
Cakes/Sweets	-0.10	0.22	0.03
Fish/Seashells	0.19	0.18	0.18
Multi/Whole grains	0.26	0.18	0.25
Legumes	-0.04	0.15	0.03
Sugar/Confectionary	0.13	0.14	0.14
Fermented vegetables	-0.04	0.12	0.03
Eggs	0.03	0.12	0.10
Pizza/burgers	0.02	0.12	-0.04
Coffee	0.18	0.09	0.22
Other fats	-0.18	0.07	-0.21
Vegetable oils	-0.06	0.05	-0.07
Dairy products	0.00	0.04	0.05
Salty snacks	-0.18	0.03	-0.10
Meats	-0.14	0.01	-0.26
Refined grains	-0.40	-0.04	-0.38
Processed meats	0.01	-0.08	-0.08
Other cereal products	-0.16	-0.12	-0.13
Other non-alcoholic beverages	-0.17	-0.28	-0.24
Explained variance (%)	11.0%	15.1%	12.6%
Correlations with *F/B* ratio[d,e]	0.08 (0.244)	0.24 (0.0004)	0.08 (0.222)

Faith PD, Faith's phylogenetic diversity

a. HiαDPs in the Korean adults were identified using three Reduced Rank Regression (RRR) models with the intakes of 25 food groups as predictor variables and each α-diversity index (Chao1, Shannon, and Faith's phylogenetic diversity) as a response variable in a RRR model

b. The α-diversity indices were log-transformed

c. The intakes of food groups were log-transformed and adjusted for total energy intake using the residual method

e. The *F/B* ratio was centered log-ratio transformed

d. Partial Spearman correlation coefficients (*p*-values) with the *F/B* ratio with sex, age, BMI, dietary supplement intake, physical activity, smoking status, and batch effect as covariates.

Table S6. Spearman correlations[1] between the high α-diversity dietary pattern (HiαDP)[2] and the relative abundance of *Firmicutes* and its genera in Korean adults of the NAS-IARC cross-sectional study (n=222 participants).

Phylum			Genus			
Taxa[3]	Coefficient	*p*-value[4]	Taxa[3]	Coefficient	*p*-value[4]	$p\text{-value}_{adj}$[4]
Firmicutes	0.079	0.249	*Lactobacillus*	0.228	0.0007	0.005
			Ruminococcus	0.214	0.002	0.007
			Eubacterium	0.202	0.003	0.008
			Hydrogenoanaerobacterium	-0.268	0.0001	0.003
			Desulfotomaculum	-0.257	0.0001	0.003
			Alkalibaculum	-0.249	0.0002	0.003
			Peptoniphilus	-0.248	0.0002	0.003
			Lactonifactor	-0.248	0.0002	0.003
			Acetivibrio	-0.247	0.0003	0.003
			Peptostreptococcus	-0.242	0.0003	0.004
			Pectinatus	-0.233	0.0006	0.005
			Cellulosilyticum	-0.229	0.0007	0.005
			Leuconostoc	-0.228	0.0007	0.005
			Subdoligranulum	-0.226	0.0009	0.006
			Papillibacter	-0.224	0.0009	0.006
			Staphylococcus	-0.223	0.0010	0.006
			Anaerococcus	-0.220	0.0011	0.006
			Succiniclasticum	-0.219	0.0012	0.006
			Acetitomaculum	-0.211	0.0019	0.008
			Anaerofilum	-0.210	0.0019	0.008
			Bacillus	-0.209	0.0021	0.008
			Solobacterium	-0.207	0.0023	0.008
			Desulfonispora	-0.207	0.0023	0.008
			Anaerosporobacter	-0.207	0.0023	0.008

Butyrivibrio	-0.206	0.0024	0.008
Pseudobutyrivibrio	-0.205	0.0025	0.008
Ethanoligenens	-0.204	0.0027	0.008
Erysipelothrix	-0.201	0.0030	0.008
Oribacterium	-0.200	0.0032	0.009
Enterococcus	-0.197	0.0038	0.010
Acidaminococcus	-0.194	0.0043	0.011
Catabacter	-0.191	0.0049	0.012
Propionispira	-0.188	0.0057	0.013
Gemella	-0.188	0.0057	0.013
Megamonas	-0.182	0.0074	0.017
Howardella	-0.180	0.0081	0.018
Lutispora	-0.180	0.0083	0.018
Allisonella	-0.178	0.0090	0.019
Lactococcus	-0.166	0.0150	0.031
Weissella	-0.163	0.0167	0.033
Peptococcus	-0.159	0.0200	0.039
Parasporobacterium	-0.158	0.0206	0.039
Anaerovorax	-0.155	0.0233	0.043
Christensenella	-0.152	0.0258	0.047

[1] Partial Spearman correlation analysis adjusted for sex, age, BMI, dietary supplement intake, physical activity, smoking status, and sample batch; [2] The high α-diversity dietary pattern (HiαDP) based on the Shannon index from Reduced Rank Regression (RRR) analysis was characterized by high intakes of fermented legumes, vegetables, potatoes, seaweeds, nuts/seeds, and tea and low intake of non-alcoholic beverages (e.g. carbonated and sweet beverages); [3] The relative abundance of taxa was centered log-ratio transformed; [4] *P*-values before (*p*-value) and after (p-value$_{adj}$) multiple comparison corrections using False Discovery Rate (FDR);

Table S7. Spearman correlations[1] between intakes of main contributing food groups[2] of the high α-diversity dietary pattern (HiαDP)[2] and the relative abundance of genera within the *Bacteroidetes* and *Firmicutes* in Korean adults of the NAS-IARC cross-sectional study (n=222 participants).

Food groups	Genus			
	Taxa[3]	Coefficient	*p*-value[4]	*p*-value$_{adj}$[4]
Fermented legumes	*Eubacterium*	0.205	0.003	0.037
	Coenonia	-0.224	0.001	0.037
	Allisonella	-0.214	0.002	0.037
	Lactonifactor	-0.211	0.002	0.037
	Hydrogenoanaerobacterium	-0.207	0.002	0.037
	Pseudobutyrivibrio	-0.203	0.003	0.037
	Peptoniphilus	-0.201	0.003	0.037
Vegetables	*Lactobacillus*	0.227	0.0008	0.070
Potatoes	*Eubacterium*	0.203	0.0028	0.109
	Lactonifactor	-0.204	0.0026	0.109
Seaweeds	*Megamonas*	-0.273	0.0282	0.132

[1] Partial Spearman correlation analysis adjusted for sex, age, BMI, dietary supplement intake, physical activity, smoking status, and sample batch; [2] The high *α*-diversity dietary pattern (Hi*α*DP) based on the Shannon index from Reduced Rank Regression (RRR) analysis and main contributing food groups of Hi*α*DP were defined based on factor loadings (> 0.3); [3] The relative abundance of taxa was centered log-ratio transformed; [4] P-values before (*p*-value) and after (*p*-value$_{adj}$) multiple comparison corrections using False Discovery Rate (FDR);

Table S8. General characteristics and lifestyle factors by three enterotypes in Korean adults of the NAS-IARC cross-sectional study (n=222 participants)

	**Weighted UniFrac**							**Bray curtis**						
	Provotella (n=64, 28.8%)		*Bacteroides* (n=81, 36.5%)		*Ruminocuccus* (n=77, 34.7%)		*p*-value[b]	*Provotella* (n=71, 25.7%)		*Bacteroides* (n=57, 32.0%)		*Ruminocuccus* (n=94, 42.3%)		*p*-value[b]
Sex – men (n, %)	32	50.0%	43	53.1%	33	42.9%	0.42	34	47.9%	32	56.1%	42	44.7%	0.39
Age (yr)[a]	29.3	22.0-37.5	29.2	22-37	30.3	22-35	0.77	30.4	42.7%	27.0	47.3%	30.6	32.6%	0.08
BMI (kg/m^2)[a]	23.3	21.0-24.9	23.1	21.1-24.5	22,4	20.6-24.2	0.12	23.3	32.8%	22.2	38.9%	23.1	24.6%	0.05
Dietary supplement – yes (n, %)	21	32.8%	29	35.8%	26	33.8%	0.76	24	33.8%	11	19.3%	41	43.6%	0.02
Physical activity – yes (n, %)	27	42.2%	32	39.5%	33	42.9%	0.90	29	40.8%	22	38.6%	41	43.6%	0.83
Smoking status – never (n, %)	44	68.8%	60	74.1%	64	83.1%	0.13	48	67.6%	42	73.7%	78	83.0%	0.07

[a.] Mean and IQR (interquartile range)

[b.] Differences in each variable among three enterotypes examined by general linear models (GLMs) for continuous variables (age and BMI) and by chi-square tests for categorical variables (sex, dietary supplement, physical activity, and smoking status)

Figure S1. Plots to select optimal numbers of clusters with (a) Elbow, (b) Silhouette, and (c) Gap statistic methods on k-means clustering for identifying enterotypes based on A) unweighted and B) weighted UniFrac, and C) Bray-Curtis distance matrices.

A. Unweighted UniFrac (determined number of clusters =2)

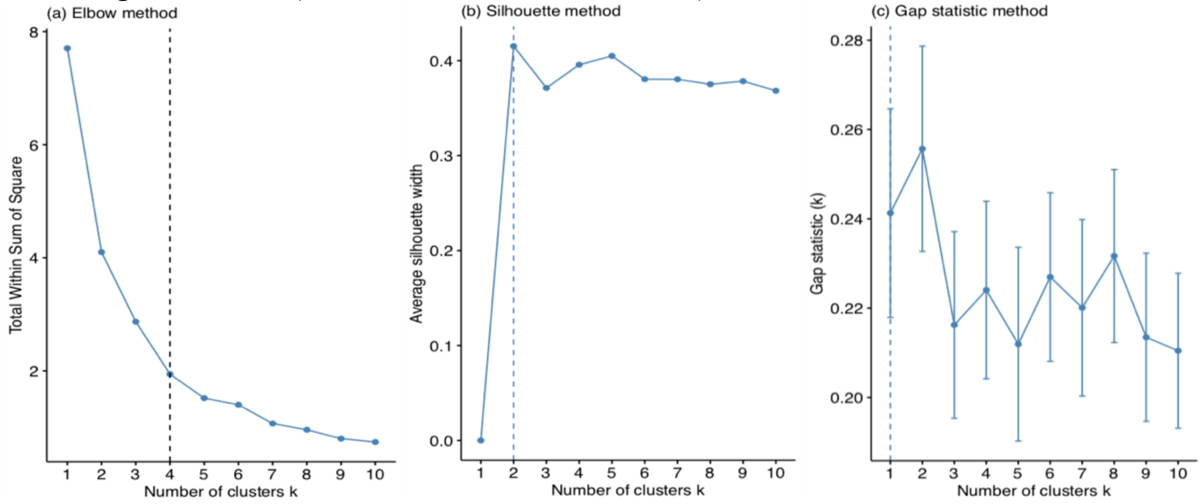

B. Weighted UniFrac (determined number of clusters =3)

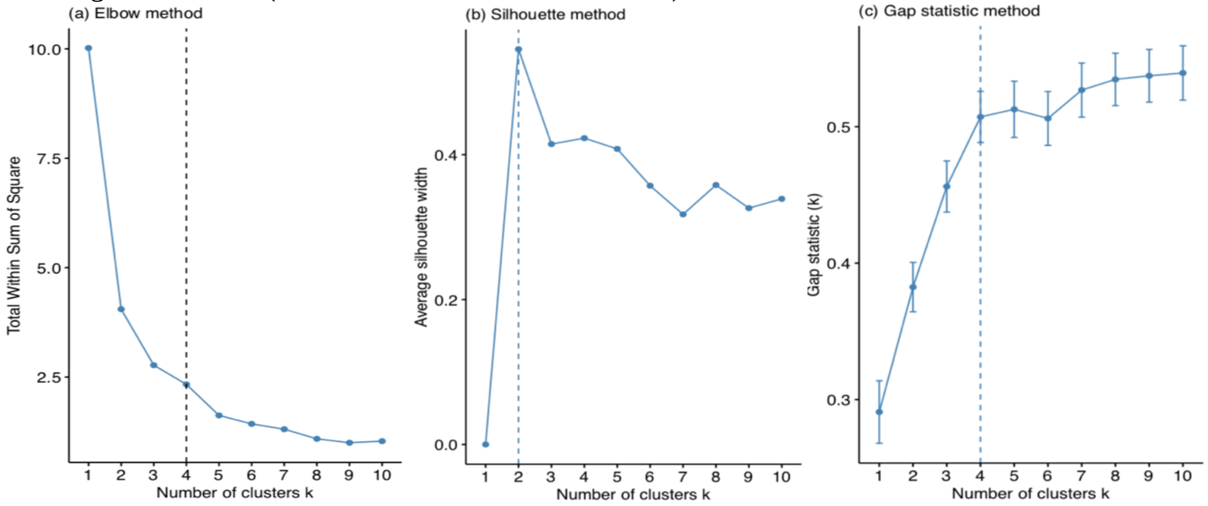

C. Bray-Curtis (determined number of clusters =3)

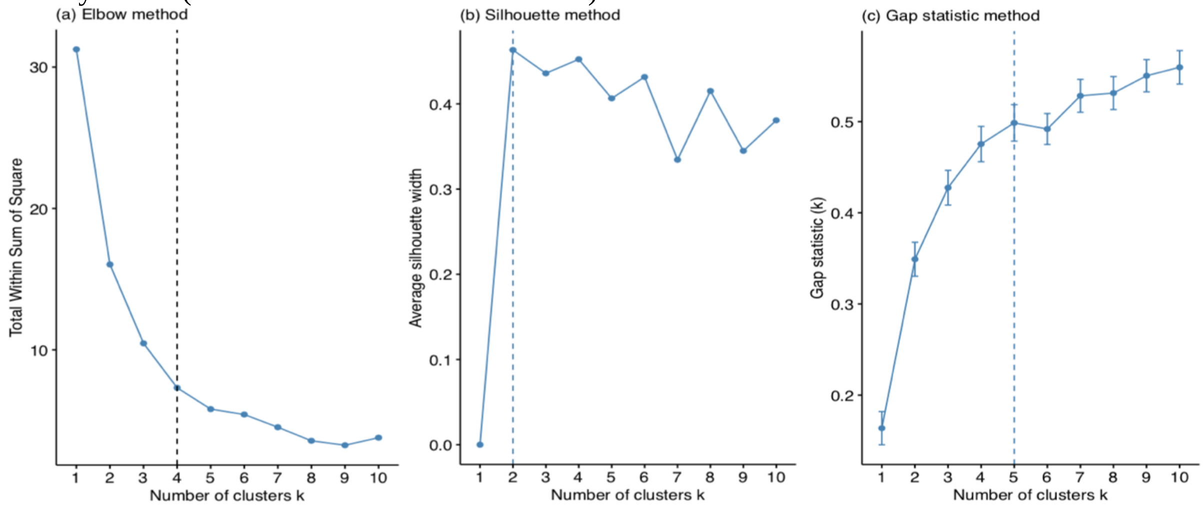

Figure S2. Enterotypes of gut microbiota in Korean adults of the NAS-IARC cross sectional study (n=222 participants) based on unweighted UniFrac. A. two enterotypes were identified by Principal Coordinates Analysis (PCoA) and k-means clustering based on unweighted UniFrac distance matrix (β-diversity) of gut microbiota in the Korean adults. B. dominant bacteria genus of each enterotype – *Bacteorides* and *Prevotella*.

A. Enterotypes based unweighted UniFrac

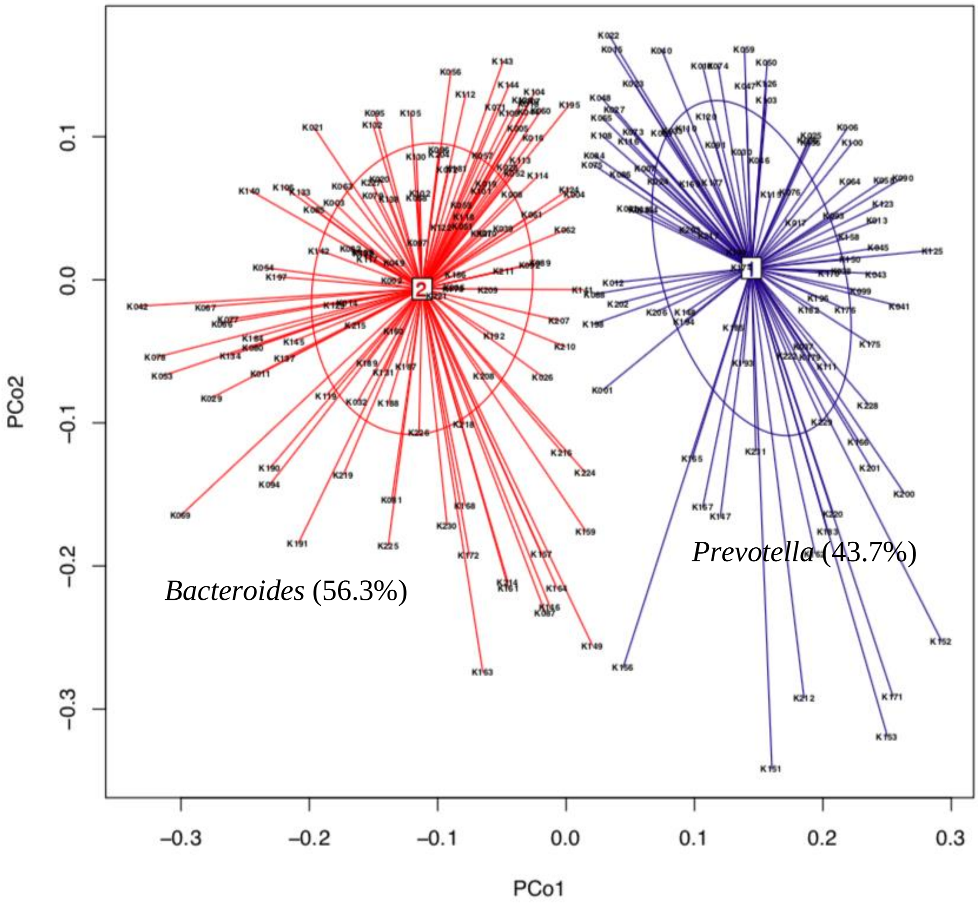

B. Dominant bacteria genus of each enterotype

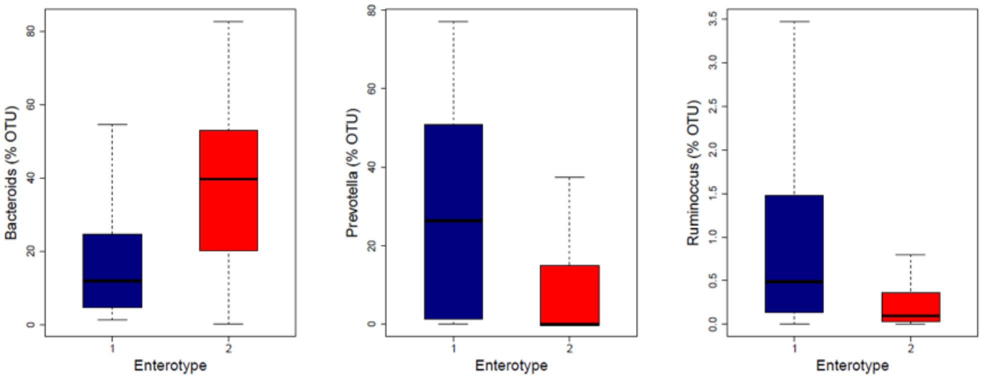

Supplement: Supplementary file 1 [file nutrients-13-00366-s001.pdf]
